# Human Papilloma Virus: An Unraveled Enigma of Universal Burden of Malignancies

**DOI:** 10.3390/pathogens12040564

**Published:** 2023-04-06

**Authors:** Ishrat Khan, R Harshithkumar, Ashwini More, Anupam Mukherjee

**Affiliations:** Division of Virology, ICMR-National AIDS Research Institute, Pune 411026, India

**Keywords:** epidemiology, disease burden, human papilloma virus, cervical cancer, oropharyngeal cancer, vaginal cancer, colorectal cancers, HPV associated malignancies

## Abstract

HPV, or Human Papilloma Virus, has been the primary causative agent of genital warts and cervical cancer worldwide. It is a sexually transmitted infection mainly affecting women of reproductive age group, also infecting men and high-risk group individuals globally, resulting in high mortality. In recent years, HPV has also been found to be the major culprit behind anogenital cancers in both gender and oropharyngeal and colorectal cancers. Few studies have reported the incidence of HPV in breast cancers as well. For a few decades, the burden of HPV-associated malignancies has been increasing at an alarming rate due to a lack of adequate awareness, famine vaccine coverage and hesitancy. The effectiveness of currently available vaccines has been limited to prophylactic efficacy and does not prevent malignancies associated with post-exposure persistent infection. This review focuses on the current burden of HPV-associated malignancies, their causes and strategies to combat the growing prevalence of the cancers. With the advent of new technologies associated with treatment pertaining to therapeutic interventions and employing effective vaccine coverage, the burden of this disease may be reduced in the population.

## 1. Introduction

**Figure 1:**
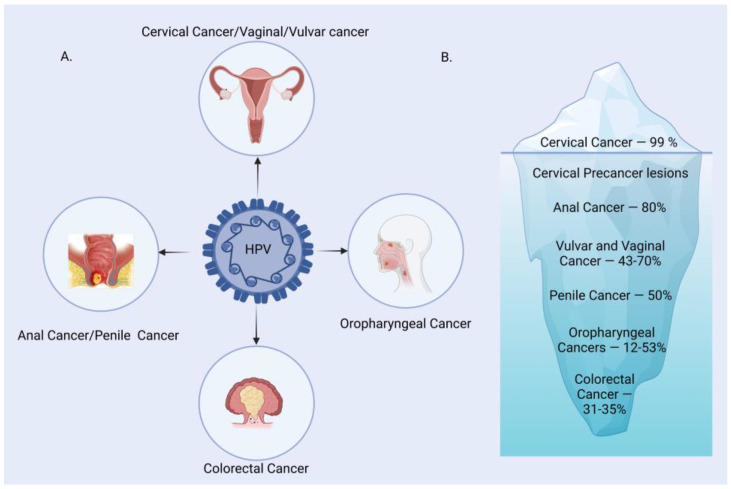
HPV-associated malignancies and their prevalence. (**A**) A major group of malignancies attributed to HPV. (**B**) Burden of HPV-related malignancies.

Human Papilloma Virus (HPV) is one of the most common causes of sexually transmitted infections worldwide. It is the main etiological agent responsible for multiple types of cancers, primarily cervical cancer in women. HPV is a small, non-enveloped, double-stranded DNA virus infecting the skin and mucosal surfaces. It is classified into two categories: low-risk HPVs (Lr-HPVs) and high-risk HPVs (Hr-HPVs). Lr-HPV is responsible for the manifestation of anogenital and cutaneous warts, while Hr-HPV is majorly responsible for oropharyngeal (oral, tonsil and throat areas) cancers and anogenital cancers, including cervical, anal, vulvar, vaginal and penile cancers [1,2,3,4,5]. In most individuals, the HPV infection clears by itself without ever developing clinical manifestations. Thus, very few of them progress to invasive cancers depending upon the type of HPV variants. The disease is often characterized by subcutaneous and anogenital warts, which may or may not develop into malignancies. The causal association between HPV and cancer has been established for many squamous cell type carcinomas, with growing evidence to link it to cervical (99%), anal (88% of cases), penile (50%), vaginal (70%), vulvar (43%), colorectal (35%) and oropharyngeal (12–53%) cancer (Figure 1) [1].

Globally, Cervical cancer is the primary cause of cancer-related mortality among women of reproductive age. Vaccination against HPV is the safest and the most reliable means of primary prevention of cervical carcinoma. Currently, there are three major vaccines available worldwide for HPV; Cervarix, Gardasil and Gardasil 9. But due to the high cost, these vaccines are not accessible to women in developing countries. The age-standardized incidence ratio for cervical cancer in the world is 14 per 100,000 women, while in India, it is 22, which is significantly higher in comparison [6,7]. More than 80 countries have introduced HPV vaccination in their national immunization programs, of which 33 are low- and middle-income countries (LMICs) [8]. Cost-effectiveness studies on HPV vaccination have shown that spending on HPV vaccinations is more cost-effective than treating cervical cancer [9,10]. Apart from cervical cancer, HPV has emerged to be one of the main causative reasons for HPV-induced other malignancies like oropharyngeal, anogenital, colorectal and breast cancers. Current guidelines are mainly focused on cervical cancer screening, but not much progress has been made toward screening for HPV-induced other cancers [11,12]. HPV-associated malignancies have different characteristics compared to other prevalent cancers in terms of variations in clinical, pathological, molecular and epidemiological profiles. As a result, their response towards therapies is markedly different and shows a better response in terms of survival rate [13,14,15]. The incidence and prevalence of HPV-associated malignancies vary remarkably in pathology and disease manifestation depending upon the HPV genotype, vaccine coverage, awareness and geographical and regional conditions [16]. Despite the improvement in vaccination coverage and other intervention studies, the high rate of morbidity related to HPV and HPV-induced cancers still persists. The worldwide burdens of HPV-associated malignancies keep increasing, and the major challenges causing the increase in the burden are associated with (A) incomplete immunization coverage due to lacunae in the established programs or programs not being followed or established successfully and (B) the time of vaccination, which usually is given late and/or not accepted among the populace. Additionally, the variability in the Hr-HPV subtypes and the resulting antigenic protection limit of current vaccines add to the already high burden of infections. These vaccines provide coverage against 7 Hr-HPV, offering 89.6% protection. The high prevalence of other viral and bacterial diseases (epidemiologic cluster) like HIV, EBV and Bacterial Vaginosis also increases the burden associated with HPV infection and HPV-associated diseases in developing countries. Also, the worldwide vaccine uptake is limited, with only a few countries reaching vaccination coverage of 90% of the adolescent female population, which is the 2030 goal of the WHO Cervical Cancer Elimination Strategy [17]. These estimates and analysis make it imperative to study the global burden of HPV and associated malignancies to combat this rising public health concern.

## 2. Burden of HPV Infection

It is crucial to have a clear epidemiological knowledge of the distribution of cervical human papillomavirus infection in the general population. This knowledge is important to devise vaccine strategies and assess the case burden of HPV over a period of time. Genital HPV infection is one of the most common sexually transmitted infections worldwide. On the basis of cross-sectional observations and meta-analysis done in 2007, it was found that approximately 10% of women worldwide with normal cytological findings through traditional methods carry a detectable cervical HPV infection, although a broad range of estimates (6.1–35.5%) has been documented, depending on the HPV testing technology, study size, the age groups and geographical region studied [18,19].

A second meta-analysis done in 2010 among 100 thousand women from 59 countries found the prevalence of HPV infection among women with normal cytological findings to be 11.7% (adjusted) [20]. The data estimated on the basis of age showed, in all the regions, a peak in HPV infection at younger ages (25 years), declining to a plateau in middle age [20]. Although this data varied on the basis of geographical locations, it gives a clear idea that during the young adult age, the possibility of acquiring HPV infection is much higher compared to other age groups. Though the vaccine-targeted types 16 and 18 are the common variant worldwide, with HPV-16 being the most common one, the other HPV types like 31, 33, 35, 39, 45, 51, 52, 56, 58 and 59 are also frequently detected in the general female population worldwide, making for 70% of HPV infections in normal cytological findings [20]. In a study conducted in 2017 in India, the overall prevalence of HPV infection was 60.33%. Out of which, the prevalence of HPV infection was 93.80% (197/210) in invasive cervical cancer (ICC) cases, 54.32% (88/162) in inflammatory smears and 19.11% (13/ 68) in normal cervical cytology. The most prevalent genotype was found to be HPV-16 (87.28%), followed by HPV-18 (24.56%) and HPV-51 (3.46%) [21].

## 3. HPV and Associated Malignancies

**Figure 2:**
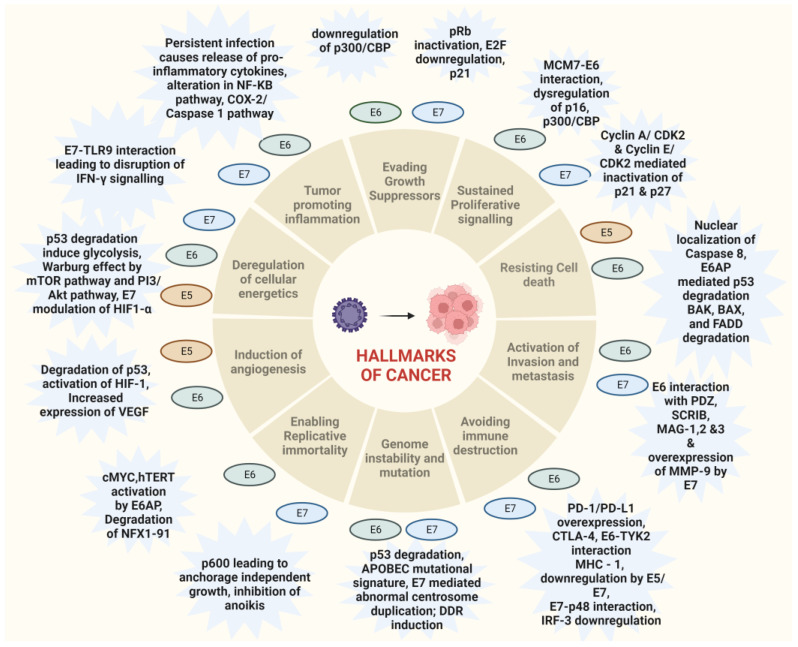
Hallmarks of cancer associated with HPV-driven oncogenic process. The major oncoproteins E6 and E7 and their interactions with specific proteins result in the modulation of major pathways associated with carcinogenesis and drive the hallmarks of cancer.

HPV-associated malignancies commonly manifest in the form of cervical, vulvar, anal, colorectal and oropharyngeal cancer. Nearly all cases of cervical cancer can be attributed to HPV infection. Although most HPV infections clear up on their own and most pre-cancerous lesions resolve spontaneously, there is a risk for all women that HPV infection may become chronic and pre-cancerous lesions progress to invasive cervical cancer. HPV being the oncogenic virus, modulates most of the hallmarks of cancer (Figure 2) [22]. HPV oncoproteins play a major role in the progression of malignancies. The hallmarks of cancer comprise eight biological capabilities along with two enabling factors acquired during neoplastic progression. HPV oncoproteins E1 and E2 play a significant role in the initiation and regulation of HPV infection. The E4 protein is majorly involved in viral release, transmission and post-translational modifications [23]. E5, E6 and E7 are major oncoproteins involved in cellular proliferation, invasion and metastasis, cell cycle arrest, angiogenesis, resisting cell death, tumor-promoting inflammation, genomic instability, evading growth suppressors, deregulation of cellular energetics, avoiding immune destruction and enabling replicative immortality [22,23]. The brief role of HPV oncoproteins responsible for their role in acquiring biological capabilities of cancer hallmarks is shown in Figure 2 [23,24,25,26,27]. The prevalence, screening, diagnosis and treatment for all types of malignancies differ in the type of organ affected. Hr-HPVs express E6 and E7, two major oncoproteins, which are responsible for the inhibition of p53 and pRB proteins in human keratinocytes and cellular immortalization (Figure 2). p53 and pRB proteins are responsible for the regulation of many key signaling pathways and gene expression [28]. HPV-associated malignancies differ from their non-HPV counterparts. The unique features of most HPV-positive cancers are summarized earlier [29]. The differences are majorly related to increased mutation activity associated with Apolipoprotein B mRNA Editing Catalytic Polypeptide-like (APOBEC) family of proteins, disrupted DNA repair pathways and altered tumor microenvironment, although it has been interesting to note that HPV-positive malignancies have better prognostic outcomes compared to their negative counterparts (Table 1) [29]. The pathogenesis of HPV-independent carcinoma is basically attributed to specific mutations in the genome. In the case of HPV-negative cervical carcinoma, HPV-negative tumors are characterized by distinct molecular profiles and lower proliferative abilities, p53 immunostaining, decreased expression of p16, p27 and p14 and alterations in PTEN, p53, KRAS, CTNNB1, ARID1A and ARID5B along with lower expression of inflammatory associated genes which explain their poor response rate to checkpoint inhibitor-based immunotherapy such as PD1/PD-L1 inhibitors [30]. In HPV-independent head and neck cancers (HNSCC), p53 mutations and p16 deactivation play an important role in transformation [31]. The main mechanism of p16 deactivation in these carcinomas is attributed to homozygous deletions, mutations and promoter hypermethylation leading to loss of CDKN2A [32]. However, p16 upregulation in HPV-negative head and neck cancers makes them difficult to differentiate them from HPV-positive cancers associated with p16 up or downregulation. Comprehensive molecular profiles of HPV-independent vulvar squamous cell carcinoma also reported alterations in TP53, TERTp, CDKN2A, CCND1 and EGFR [33].

## 4. Cervical Cancer

Cervical cancer is the main cause of cancer-related mortality among women worldwide. A study conducted by Munoz et al. pooled data from 11 case-control studies to determine the association between cervical cancer and HPV infection from multiple countries; 15 HPV types have been classified as high risk, three have been classified as probable high-risk, 12 have been classified as low risk and three are considered to have an undetermined risk based on the progression to cervical cancer [34]. The current method widely used for screening is traditional PAP smear-based cytological analysis which is invasive, time-consuming and requires expertise. The increasing uptake in HPV vaccination rate and the introduction of new technologies and approaches to cervical cancer detection, such as HPV self-testing, are shifting the historical approach to screening cervical cancer [35]. As per the current guidelines in different countries, HPV-DNA tests are used for women over 25 years old, and a PAP test is only done if the HPV-DNA test gives a positive result [36,37,38,39,40,41]. A randomized study examined the effect of the HPV-DNA-based cervical screening method and found it to be more effective than the Pap-Test in preventing cervical cancer as it allows early, rapid and accurate identification of high-grade persistent lesions and low-grade lesions [42]. The high-risk genotype HPV-16 and HPV-18 has the highest chances for neoplastic progression, accounting for over 50% and 20% of cervical carcinoma cases, respectively [5]. A number of risk factors are associated with HPV infection and, thereby, progression to cervical cancer. The major risk factors include reproductive and sexual elements, behavioral aspects etc. The highest incidence of cervical cancer is observed among patients reporting sexual intercourse at a young age (<16 years old), multiple sexual partners, smoking and low socio-economic level [43,44]. Infection with other STIs (Sexually Transmitted Infections) like HIV also poses a greater danger towards HPV infection. The risk of developing infection from high-risk HPV types is higher in women with HIV [45,46,47,48,49]. The results of the studies conducted to evaluate the relationship between HIV and cervical cancer suggested a higher rate of persistent HPV infection with multiple oncogene viruses, higher abnormal PAP smears and a higher incidence of high-grade intraepithelial lesions (HSIL) among people with HIV [46]. Apart from that use of oral contraceptive pills for more than 5 years can double the risk of cancer [47,49].

**Figure 3:**
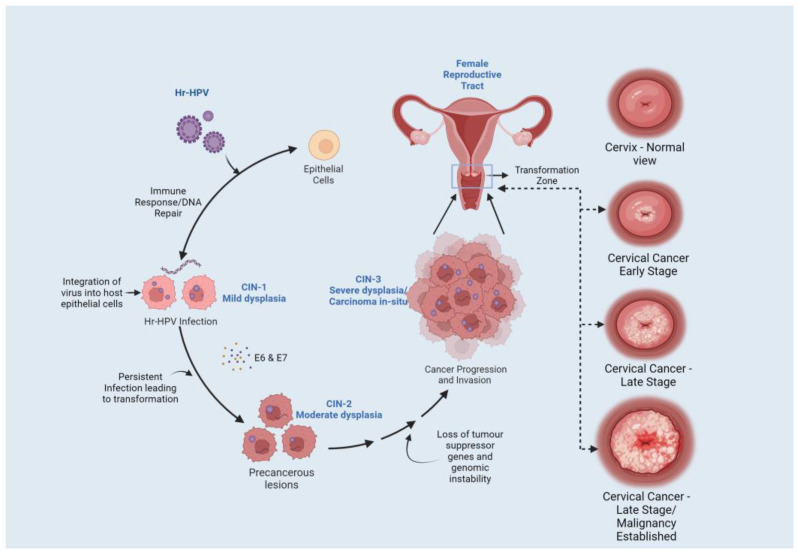
HPV Infection and Cervical Cancer Progression. Infection with Hr-HPV contributes to the initiation of the immune response; however, due to persistent infection and interaction of E6 and E7 oncoproteins affecting various molecular pathways results in the formation of pre-cancerous lesions and subsequent progression to cervical carcinoma.

Persistent infection of HPV leads to the progression of cervical carcinoma (Figure 3). HPV causes the immortalization of cells and promotes cervical dysplasia (also called cervical intraepithelial neoplasia or CIN). Cervical dysplasia subsequently progresses to mild, moderate and severe dysplasia and invasive cervical cancer. The immortalization of HPV-infected cells is caused due to E6 & E7 oncoproteins-regulated apoptosis and cell cycle [50]. The inactivation of p53 and pRb is predominantly associated with HPV-associated cervical cancer. The inactivation of p53 is caused by the E6 oncoprotein and E6-associated protein (E6AP) complex, which is an E3 ubiquitin-protein ligase [51]. The tumor suppressor gene, Notch 1, induces cellular differentiation in keratinocyte cells. Notch 1 is also responsible for reducing cellular proliferation in cervical cell lines through E6 and E7 oncogenes. E6 suppresses the expression of Notch 1 through the inactivation of the p53 protein [52]. In cervical cancer, p53 degradation by E6 results in inhibiting the expression of Notch 1, leading to malignant transformations and inducing p53-mediated apoptosis [53]. Multiple studies have demonstrated the higher expression of p53 with the progression of cervical cancer [54,55].

**Figure 4:**
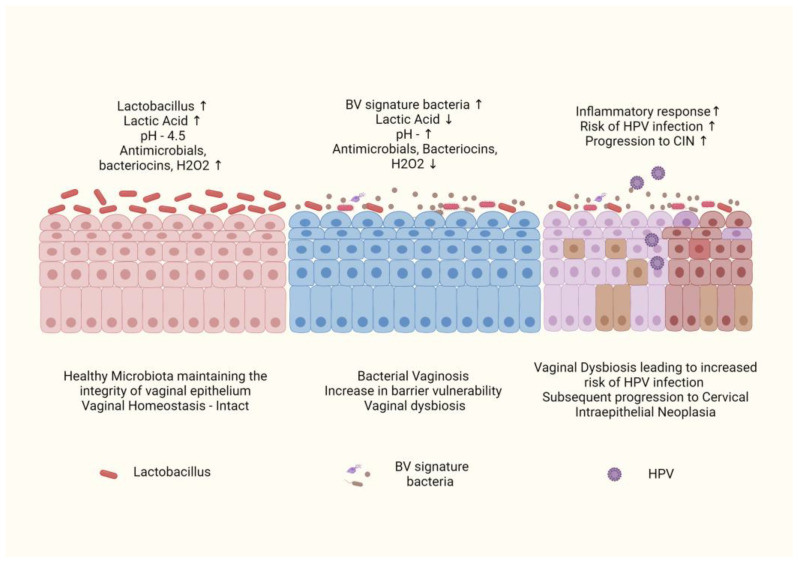
Role of Vaginal microbiota in HPV infection. Normal microbiota comprises *lactobacillus* that maintains acidic pH and acts as the first line of defense in the vagina. Overgrowth of BV-associated bacteria leads to vaginal dysbiosis that increases the risk of the acquisition of viral infections, including HPV.

The mucosal surface of the female reproductive system is the primary site of HPV infection. The mucosal system acts as the first line of host defense in eliminating the risks of any probable infections, including HPV [56]. Vaginal microbiota makes an important part of the vaginal ecosystem, maintaining homeostasis. The detailed composition and relative abundance of vaginal microbiota have been evaluated through 16s rRNA sequencing [57]. Five microbial community states (CST) have been characterized, out of which four CST types (CST-I, II, III and V) are dominated by *Lactobacillus* sp. (*L.crispatus*, *L. gasseri*, *L. iners* and *L. gasseri*), while CST-IV comprised higher level of anaerobic bacteria like *Gardnerella*, *Atopobium*, *Prevotella*, *Mobiluncus*, *Streptococcus*, *Mycoplasma, Sneathia* and *Ureaplasma* [57]. Vaginal dysbiosis occurs as a result of low abundances of *Lactobacillus* species and higher levels of anaerobic species, causing a disturbance in vaginal homeostasis resulting in Bacterial vaginosis (BV). The increase in cervicovaginal pH and reduction in lactic acid-producing bacteria disturbs the normal microflora and induces the growth of atypical bacterial growth. BV is known to be associated with an increased risk of HPV infection and subsequent progression to cervical intraepithelial neoplasia. BV is associated with an increase in proinflammatory cytokines and a decrease in levels of SLPI (Secretory leukocyte protease inhibitor), an anti-inflammatory cytokine that causes changes in the immune system, increasing the chances of infection with HPV. Additionally, oxidative DNA damage associated with BV results in the generation of ROS, which cause DNA breaks in the host genome and HPV episome, enhancing viral acquisition and integration and resulting in neoplastic transformation similar to that employed by the HPV E6 oncoprotein [58]. Multiple studies have shown a positive association between the abundance of normal microflora of *Lactobacillus* sp. resulting in lower chances of HPV acquisition and enhanced HPV clearance. On the contrary, an increase in anaerobic species like *Gardnerella* results in a higher rate of HPV acquisition, the persistence of HPV infection and progression to cervical carcinogenesis [59,60,61,62,63,64] (Figure 4). *L. iners* is one of the species of lactic acid-producing bacteria known to enhance dysbiosis and is correlated with a high frequency of HPV infection [58].

Apart from this, epigenetic factors like microRNAs (miRNAs) also modulate the gene expression and function in HPV-induced cervical cancer. miRNAs, a group of small non-coding RNA, are involved in the pathogenesis of cervical cancer by posttranscriptional regulation of gene expression. miRNAs in cervical cancer may act as OncomiRs or Tumor suppressor miRNAs. OncomiRs or oncogenic miRNAs regulate the function of tumor suppressor genes by inhibiting them, thereby inducing carcinogenesis, while dysregulation of Tumor Suppressor miRNAs results in the upregulation of oncogenes resulting in malignant transformations [65]. As a result, they can play a pivotal role in the diagnosis as well as treatment of cancers. Microarray-based RT-PCR analyses have identified the role of miRNAs in cervical cancer progression. Cellular miRNAs like miR-21, miR-19a and miR-19b are overexpressed in many types of cancers, including cervical cancer [66]. miR-21 is a negative regulator of PDCD4 (Programmed cell death 4). PDCD4 regulates apoptosis, blocks translation and inhibits tumor growth. miR-21, when interacting with PDCD4, induces cell growth [67]. miR-19a and 19b increase cell differentiation and proliferation, in vitro, in Hela and C-33a cell lines [66]. Oncogenic miRNAs like miR-9, miR-10, miR-205 etc., promotes cell invasion, proliferation, migration and metastasis by dysregulating target genes, while tumor suppressor miRNAs like miR-26a, miR-126, miR-132 etc., inhibits these hallmarks of cancers [66]. Chronic inflammation is also one of the main precursors of cervical carcinogenesis, which is characterized by the production of ROS, cytokines, growth factors and enzymes leading to malignancies. Persistent HPV infection induced by E6 and E7 oncoproteins results in the modulation of the NF-κB pathway to promote inflammation. The activation of NF-κB mediated COX2/Caspase-1 signaling pathway has been known to induce cell proliferation, inflammation and apoptosis in cervical cancer [68,69].

## 5. Oropharyngeal Cancer

**Figure 5:**
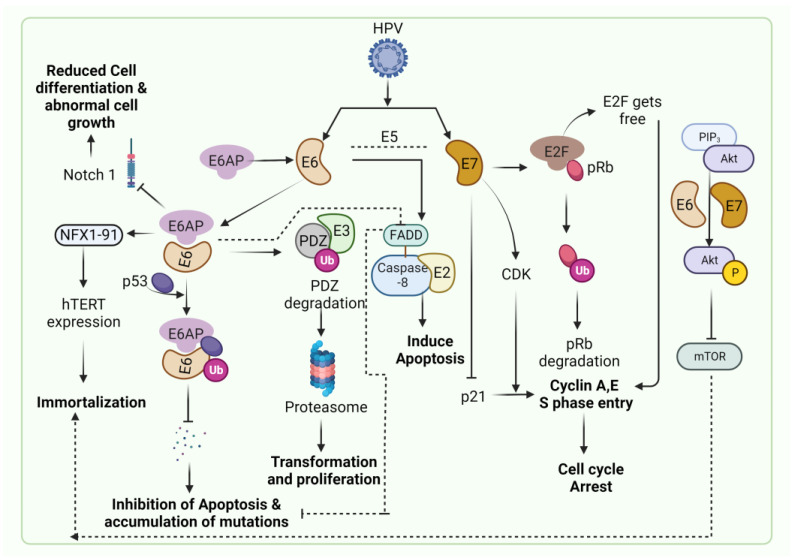
Common pathways associated with HPV-induced malignancies. E6 and E7 oncoproteins mediate p53 and pRB degradation via ubiquitin-dependent proteosome degradation of p53 and pRB inactivation. E6 binds to the LxxLL motif of E6AP, leading to the degradation of p53, causing evasion of cell death, inhibition of apoptosis and accumulation of mutations. E6 also targets other apoptotic signaling cascade molecules like FADD and NFX1-91 to induce apoptosis and immortalization of malignant cells, respectively. E6-E6AP downregulates Notch 1 through the inactivation of p53, leading to reduced cell differentiation and abnormal cell growth. E7 causes deregulation of CDK by inactivation of p21 resulting in cell cycle arrest. The PIP/Akt pathway activation by HPV E6 and E7 results in immortalization.

Head and neck cancer is the sixth most commonly diagnosed cancer. It is majorly attributed to smoking habits and the use of tobacco. However, recent studies have demonstrated HPV as one of the main causes of oropharyngeal cancer, the prime suspect being the HPV-16 genotype [70]. HPV-DNA detection in oropharyngeal tumors increased from 16.3% during the period from 1984 to 1989 to 71.7% during the period from 2000 to 2004 [71]. Studies showed that repression of viral oncogene expression in HPV-positive oropharyngeal cancer cells induces massive apoptosis and restoration of p53 and pRb tumor suppressor pathways (Figure 5) [71,72]. HPV-induced head and neck cancer is caused by the wild-type TP53 that activates PIK3CA mutation, lower EGFR expression and upregulation of tumor suppressor p16, which is also used as a biomarker for this cancer [73,74,75]. HPV-associated oropharyngeal cancer shows distinct differences from its other counterparts, where smoking and tobacco use is the key player [76].

Among all cancers, oropharyngeal squamous cell carcinoma or OPSCC has one of the most rapidly rising incidences in high-income countries [77,78]. In 2021, globally, the prevalence of oropharyngeal cancer was reported to be 33%; however, prevalence varies considerably depending on the geographical region, with estimates ranging from 0% in southern India to 85% in Lebanon [79]. In general, screening and diagnostic strategies involve PET, MRI and HPV DNA detection among patients [24,80,81].

## 6. Vulvar and Vaginal Cancer

Hr-HPV is always considered to be the major contributor to cervical cancer, likewise, with growing evidence for oropharyngeal and colorectal cancer. However, the relationship of HPV with vulvar and vaginal cancers is still debatable and lacks contemporary information about the prevalence of HPV-associated malignant transformations, its precursor lesions and the identification of HPV status in the prognosis of malignancy.

Vulvar squamous cell carcinoma, or VSCC, is typically a rarer form of malignancy, accounting for 4–6% of all gynecological cancers [82]. There are two pathways associated with vulvar cancers; the first pathway is attributed to underlying autoimmune responses and is HPV-negative, most likely resulting from TP53 mutations, predominantly occurring in older women above 69 years of age. At the same time, the second one is caused by the mucosal Hr-HPV infection, which is persistent over time and most commonly occurs in young women [83,84]. The estimates of HPV prevalence in the vulvar intraepithelial neoplasia were 84%, as reported in a meta-analysis in 2009 [85]. Few studies reported favorable outcomes with respect to the presence of HPV DNA in the tumor tissue and found it to be an independent prognostic factor in VSCC [86,87]. Hr-HPV infection has been identified as common in vulvar intraepithelial neoplasia (VIN), but the detection of Hr-HPV was associated with the reduction in risk of progression to malignancy and improved survival with VSCC [88]. However, recent evidence provided contrasting insights into the incidence and progression of VSCC in younger ages. In some countries, the incidence of VIN increased four-fold between 1973–2000 in the younger population [89,90]. This might be due to the increase in Hr-HPV-associated infections with the onset of sexual activity, changes in sexual behavior and increased transmission. Currently, the HPV-associated vulvar cancer global burden stands at 43% [1]. Though HPV-associated vulvar cancer progress slowly compared to HPV-negative cancers, they pose an increased risk of developing into additional HPV-associated lesions of the anogenital tract.

Vaginal cancer is a rare form of malignancy, mostly resulting the metastatic invasions from other sites [91]. Overall, this type of cancer accounts for approximately 1–2% of cases among all gynecological cancers [92]. Vaginal cancers predominantly occur in elderly women and post-menopausal women. With the increase in the prevalence of HPV infections over the last decade, vaginal cancer is reported in the younger population too. The persistent HPV infection leading to malignant transformations from cervical cancer results in metastasis in the vagina [93]. HPV-16 is found to be the possible cause of HSIL and carcinoma of the vagina [94,95,96]. A recent meta-analysis study conducted, including 26 different studies, found the prevalence of HPV-associated vaginal cancer at 67% [97]. The pathways allied with HPV-associated malignancy in the vagina are similar to the basic pathway involving E6 and E7 oncoproteins. However, the role of HPV as a prognostic factor with respect to vaginal cancer remains sparse. The role of p16 and p53 as prognostic factors to analyze the malignant potential in vaginal cancer has not been effectively evaluated and studied as compared to the other HPV-associated malignancies. Recently, a systematic review was done comprising 12 different studies to summarize the prognostic value of HPV, p53 and p16 on vaginal cancer [98]. This review reported that women with HPV and p16-positive vaginal cancer had improved prognoses as compared to HPV-negative and p16-negative vaginal cancer [98]. Vaginal cancer is primarily linked to the HPV-16 subtype; however, in one of the unusual case studies, HPV-68 was found to have the potential oncogenic effect in HIV-positive women leading to vaginal squamous cell carcinoma. The study also demonstrated that a normal cervix could have HPV negative test, whereas vaginal carcinoma can be HPV positive [99]. These findings report that Hr-HPV contributes to the vaginal cancer burden, but more studies are required to understand the possible impact.

## 7. Anal Cancer

Anal cancer is a rare form of malignancy, accounting for less than 5% of all gastrointestinal cancers [100]. They are thought to be more common in women compared to men; however, recent years have seen an increase in incidence and prevalence in both genders. The incidence is higher in people living with HIV, other STIs and MSM (men who have sex with men), and the risk is higher among women living with HIV [101,102]. HPV-16 is the causative agent for around 90% of anal cancers caused by Hr-HPV subtypes [103]. In HIV-positive patients, immunosuppression is attributed to the increased risk of acquiring HPV infection and subsequent progression to anal cancer [104,105]. The high prevalence of Hr-HPV anal infections (40.1%) and high risk of abnormal cytological lesions of the anus (39.5%) are found in women living with HIV exhibiting HIV as the major factor associated with HPV infections and anal cancers in women [106]. Some studies have also shown that the chances in women increase with a history of high risk of cervical cancer or cervical lesions and are more prone to developing anal cancers in a decade after diagnosis with an Odds ratio (OR) value of 10.5 and 13.6 [107,108]. It was also reported that the prevalence of anal cancer increases according to cervical lesion grade, with the occurrence of 15% in low-grade squamous intraepithelial lesions (LSIL) and 55% in microinvasive carcinoma [109]. However, in another study, it was reported no significant association between LSIL vs. HSIL/CC to anal HPV infection [110]. This suggests that there are multiple risk factors associated with the progression of anal malignancies along with HPV infections, which need to be explored further.

## 8. Colorectal Cancer

Colorectal cancer (CRC) is one of the common malignancies occurring predominantly in the male population in the world. Multiple risk factors owing to the progression of malignancy in CRC, like genetic predisposition, have been documented, but the molecular mechanisms associated with the same have not been fully explored yet. The association between HPV infections and CRC has been controversial and inconclusive, owing to the different outcomes obtained in multiple studies. Previous reports have shown a positive association between HPV infection and the increased risk of CRC [111,112,113]. Some of the studies have reported a high prevalence of HPV DNA in malignant tissues compared to the healthy controls [114,115]. There are several reports involving the detection of HPV DNA in CRC have pointed out that HPV may be the cause of carcinogenesis even though the prevalence of HPV in CRC is not common [116,117,118,119]. The studies, which have not found any correlation of HPV DNA or virus-like particle antibodies in CRC samples, challenged the positive association between HPV infection and CRC [120,121,122,123]. Overall, the prevalence of HPV DNA in CRC samples has been found to be 0–84%, which is a vast range [124]. It was found that the HPV DNA is commonly present in malignant colorectal tissues but not in its noncancerous counterpart or peripheral blood, indicating the role of HPV pathogenesis in colorectal cancer [125]. The mean value of the prevalence of CRC is 41.7% compared to 32% in adjacent non-cancerous tissues [114]. CRC is characterized by the mutation in p53 genes, but in the tissues infected with HPV, the TP53 gene is found to be intact. However, the disrupted expression of this gene might be leading to carcinogenesis due to the inactivation of p53 by HPV oncoproteins [126]. Additionally, the modulation in the Wnt/β catenin pathway due to the pro-oncogenic effects of HPV is also one of the factors suggesting a possible association between HPV infection and CRC [127]. E6 protein modulates β catenin pathway and increases the rate of translocation to the nucleus, while E7 binds to HDAC (histone deacetylase) and E2F suppressor p21, resulting in the elongation of S-phase (Figure 5) [128,129,130]. This modulation results in genomic instability and an increased rate of foreign DNA integration into the transfected cells [131,132]. The extension of the S-phase and delay in DNA replication results in the activation of p53, which triggers apoptosis [133]. HPV causes ubiquitin-dependent degradation of p53 by encoding E6, which results in the disruption of cell cycle checkpoints [134,135,136]. Hence, it is clearly identified that there might be a significant relationship between HPV and CRC in the way it modulates the function of various genes and pathways associated with CRC [137]. A recent meta-analysis comprising a total of 2937 patients out of which 1562 (53.2%) were diagnosed with CRC and 1375 (46.8%) control samples, HPV was detected in 424 (27.1%) CRC samples. In the healthy tissue specimens, 129 (9.4%) were found to be positive for HPV. Overall, it was observed that HPV positive patients have a six-times greater chance of developing the malignancies associated with CRC when compared to HPV negative patients (OR = 6.398, CI (95%) = 3.025–13.533; *p* < 0.0001) [138]. This data is quite significant in drawing the relationship between HPV and CRC.

## 9. Other Cancer Types Associated with HPV

Apart from the major cancers discussed above, HPV is known to be linked to breast cancer, penile cancer, ovarian cancer, renal adenocarcinomas, lung cancer, liver and even brain, although the studies associated with them are relatively fewer, and the mechanism behind HPV mediated malignant transformations in these malignancies remain elusive.

Breast cancer is the most common cancer type among women worldwide, attributing to the highest cancer-related mortality. According to estimations, more than 2 million new cases of breast cancer and 684,996 deaths were recorded to this malignancy [139]. Recently, some other studies also reported the association between HPV infections in breast cancer. However, the frequency of HPV-associated breast cancer varied widely in different studies (1.6-86%) [140,141]. To date, nine different HPV types (HPV-6, -11, -16, -18, -31, -33, -35, -45 and -52) have been found in breast cancer across different populations worldwide [142]. The possible route of HPV infection and its mechanism in breast cancer is not fully understood. The two main hypotheses have been proposed corresponding to HPV infection in mammary gland cells; (A) the viral transmission is mediated via the blood, lymphatic systems or other bodily fluids to the mammary glands in patients with a previous history of HPV-associated uterine and cervical dysplasia and (B) The transmission is also possible through virus spillover in the circulatory system from the primary HPV infection or tumor site as HPV DNA was found in PBMC (peripheral blood mononuclear cells) [143,144]. However, other reports contradict that HPV viremia is improbable as the HPV lifecycle occurs in the basal stratified epithelial cells [145]. An additional interesting hypothesis is based on the extracellular vesicles (EVs) mediated transmission of HPV, where the viral DNA was found to be present in serum-derived EVs from middle rectum and breast cancer patients and may transmit HPV [146]. Other routes may involve transmission via sexual activity through the micro-lesions of the breast [142]. HPV positivity has been detected in 32.42%, 12.91%, 42.9% and 15.1% of breast cancer patients from Asia, Europe, North America, Australia and Central and South America, respectively [147,148]. Several studies have also reported the presence of more prevalent HPV DNA in triple-negative breast cancer (TNBC) and HER2+ breast cancer compared to luminal types of breast cancer [149,150]. E6 and E7 oncoproteins are known to interact with BRCA1 and BRCA2 genes resulting in changes in cellular transformation pathways. These findings suggest that HPV DNA might contribute to aggressive breast tumors; however, its role in the initiation of malignancies remains to be thoroughly studied.

Penile Cancer is a rare form of malignancy contributing to 0.1–1 in 100,000 men in high-income countries [151]. It makes up to 0.5% of all cancers occurring in men [152]. The pathogenesis can be HPV-dependent or HPV-independent. HPV-associated penile squamous cell carcinoma manifests as warty or basaloid features and progresses to penile intraepithelial neoplasia [153]. HPV DNA has been detected in 14–100% of invasive carcinoma of the penis based on the method of detection [154]. The mechanism behind HPV-associated progression to malignancy in penile cancers remains to be studied extensively.

Ovarian cancer, though rare, is a life-threatening malignancy occurring in women. The association between ovarian cancer and HPV infection is ambiguous. It is widely known that HPV is the main causative agent behind cervical cancer; however, its role in carcinogenesis in ovaries is under studied and conflicting. A meta-analysis published in 2021 investigated 29 studies consisting of 2280 cases of ovarian cancer and reported a 15.9% (95% CI, 11–22) pooled prevalence of HPV. The low prevalence might be due to the affinity of HPV to infect certain squamous cells of the cervix [155]. The prevalence of HPV varied markedly depending upon geographical locations, with the highest prevalence of HPV reported by studies from Asia (30.9%; 95% CI, 20–44) and Eastern Europe (29.3%; 95% CI, 4.4–78). The most frequently detected HPV genotype was reported to be HPV-16 (54%; 95% CI, 27.9–55), followed by HPV-18 (23.2%; 95% CI, 18.8–28.2) [155]. However, more studies need to be done to clearly understand the plausible role of HPV in ovarian cancer.

HPV detection in renal cell carcinomas and metastasis to the lungs, liver and brain is contradictory in various studies; however, it may be possible due to the association between major malignancies like oropharyngeal, head and neck cancer and cervical cancer metastasis with HPV [154,156,157,158]. These malignancies currently do not impart burden to the existing load of major cancers associated with HPV, although studies should be done to understand the probable cause of invasive carcinoma and the associated risk it may pose to the patients in the long run.

## 10. Vaccine Status

**Figure 6:**
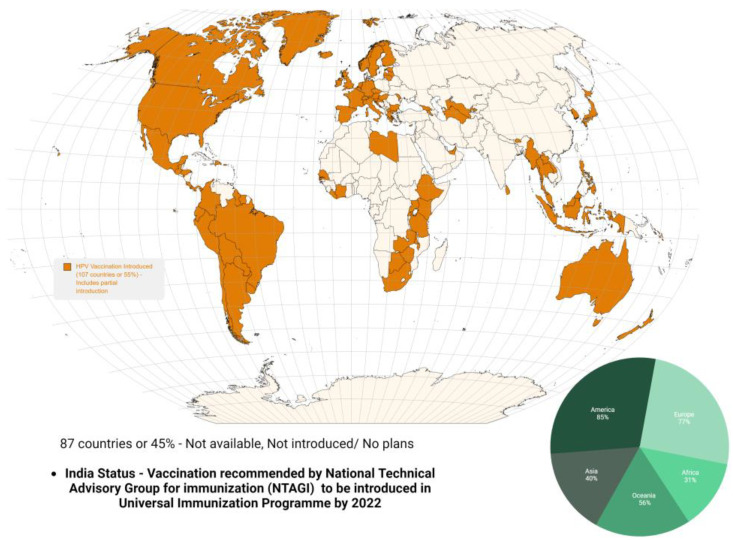
Percentage of the WHO member states countries implementing HPV vaccination program by 2020.

HPV-associated research has led to the preparation of vaccines against the HPV virus, thereby paving the way for a decrease in the burden of HPV infections and associated cancers, majorly cervical carcinoma. Currently, there are three HPV vaccines targeting two, four or nine HPV types, which account for 70–90% of HPV-related cancers. All three of these vaccines are efficacious at preventing HPV infection in randomized trials and represent one of the first primary cancer prevention approaches on a global scale. Currently, at least 118 million women have received one dose of the HPV vaccine. This number is encouraging but only represents 3.5% of the world population [159,160]. The three currently available Cervarix, Gardasil, and Gardasil 9 are prophylactic vaccines meant only for HPV exposure naïve individuals. The bivalent vaccine (Cervarix) is effective against HPV 16 and 18, and the quadrivalent vaccine (Gardasil) is effective against HPV 16, 18, 6 and 11 [161]. The nonavalent vaccine (Gardasil 9) is effective against two to nine HPV subtype strains which are HPV 6, 11, 16, 18, 31, 33, 45, 52 and 58. These vaccines are effective in reducing cervical disease and anogenital dysplasia in controlled clinical trials and studies. However, the introduction of these vaccines in universal immunization programs in developing countries is far-fetched due to their cost, limited resources, awareness and hesitancy among the population. As per the WHO data, by June 2020, 107 (55%) of the 194 WHO member states have considered introducing HPV vaccination among its population nationwide (Figure 6) [17].

HPV oncoproteins E6 and E7 are responsible for affecting the DNA damage checkpoint controls on interaction with p53 and pRb proteins resulting in unregulated growth and malignancy in the cells. The replication of HPV occurs in differentiated cells by maintaining a low copy number in basal epithelial cells. Due to this low copy number, HPV undergoes immune evasion from the host immune system and may elicit a typically weak immune response [162]. Persistent HPV infection leads to seroconversion and may or may not increase the risk of progression to precancerous lesions [163,164]. The basic approach for the reduction of HPV infection has been the development of vaccines to prevent viral acquisition and integration. Available HPV vaccines are composed of virus-like particles, basically inactivated HPV capsid protein L1, from Hr-HPV subtypes. These vaccines are known to generate robust immune responses by inducing high titers of neutralizing HPV capsid antibodies, which prevent the interaction of HPV with the host cell surface and inhibit viral entry [165,166,167,168].

There are two types of vaccines that work against viral infections; Prophylactic vaccines and Therapeutic vaccines [169,170]. Prophylactic vaccines elicit an immune response by the generation of neutralizing antibodies (especially IgG), while therapeutic vaccines work by eliciting immune responses by stimulating cell-mediated immunity (especially CD8+ T cells) [171]. Currently available bivalent, quadrivalent and nonavalent vaccines are prophylactic in approach. The nonavalent prophylactic vaccine Gardasil^®^ 9 could prevent 90% of HPV-related cervical cancer cases and 50% of all other HPV-related malignancies [172]. The main drawback of prophylactic vaccines is that they are unable to clear already established infections or precancerous lesions by mounting long-term cellular CD4+ and CD8+ immune responses [173]. Currently, as per the WHO 2022 report, 6 prophylactic vaccines are licensed to be used for the prevention of HPV-associated malignancies. The prophylactic vaccines (Table 2) provide protection against the different target strains and, therefore not able to provide protection against all HPV-related malignancies. Hence, there is a growing need for therapeutic vaccines and other cancer therapies.

E6 and E7 oncoproteins are the preferred targets for developing therapeutic vaccines owing to their role in the onset and maintenance of malignancies and higher expression in HPV infection and precancerous lesions, which is limited to the infected cells and lesions [174]. Additionally, since these oncoproteins are responsible for the transformation of malignant cells and driving the phenotype of these cells, antigen loss is not one of the mechanisms for immune evasion [175]. These properties make them ideal targets for therapeutic vaccines attacking specific tumor cells bypassing healthy cells and tissues.

**Table 2:** Prophylactic vaccines available to prevent HPV-associated cancers [176,177].

Vaccine	Type	Target Strains	Manufacturer	Licensed Year	Target Population
Cervarix^®^	Bivalent	16/18	Glaxo SmihtKline	2007	Females & Males
Gardasil^®^	Quadrivalent	6/11/16/18	Merck	2006	Females & Males
Gardasil^®^ 9	Nonavalent	6/11/16/18/31/33/45/52/58	Merck	2014	Females & Males
Cecolin^®^	Bivalent	16/18	Xiamen Innovax Biotech	2020	Females
Cervavac^®^	Quadrivalent	6/11/16/18	Serum Institute of India	2022	Females & Males
Walrinvax^®^	Bivalent	16/18	Walvax/Zerun	2022	Females

E1 and E2 are ideal targets for early viral infections as they are expressed before viral integration in the genome. Targeting these oncoproteins will be a preferable strategy to develop therapeutic HPV vaccines as the approach will generate cytotoxic (CTL) responses and strong tumor-specific T-cell responses to eliminate infected and malignant cells [178,179,180]. There are various strategies being employed for the preparation of therapeutic HPV vaccines. Live vector vaccines, nucleic acid, protein, whole cell and combination vaccines are some of the strategies being explored currently for therapeutic HPV vaccines. Live vector vaccines involve the use of bacterial and viral vectors for the introduction of HPV antigens in the host to elicit an immune response. Subunit vaccines involve the direct introduction of antigens in the form of peptides or whole proteins. Nucleic acid vaccines use DNA and RNA amplicons-based DNA vaccines. DNA vaccines are one of the safest approaches, easy to manufacture and purify and promote MHC class I antigen presentation, which makes them the ideal choice for repeated vaccination. Cell-based techniques involve dendritic cell-based and adoptive cell transfer for antigen presentation and T-cell activation. The strategy involves the transfer of dendritic cells or T cells manipulated ex vivo after the isolation from the patient and then transferring them back to elicit an immune response [161,180].

The combination of prophylactic vaccines and therapeutic vaccines also presents an effective strategy for vaccination among young women and older women who have been previously exposed to HPV infection. The combination therapy will be beneficial in generating short-term and long-term immune responses among this population. Chimeric proteins and pseudovirions are used as delivery vehicles for DNA vaccines employed in combination vaccination [181].

With the increasing burden of malignancies associated with HPV, it is necessary to develop therapeutic vaccines that will help in eliminating long-term infection and targeted approaches to the prevention of cancer. Numerous clinical trials have shown promising results in the development of therapeutic vaccines, thereby paving the way for multiple approaches to the elimination of the HPV-related cancer burden.

## 11. Alternate Therapies and Novel Therapeutics

Vaccines have proved to be an important milestone in the prevention of HPV-associated malignancies, predominantly cervical cancer. However, due to the increasing burden of these malignancies and the surging demand for therapeutic vaccines to overcome the limitations associated with prophylactic vaccines, multiple approaches are being explored to enhance the efficacy of currently available drugs and new domains like the role of epigenetic regulation, and their modulators are being researched extensively.

There is no HPV-specific antiviral treatment for the infection. The screening and treatment of cervical precancerous lesions is an effective strategy to combat cervical cancer. The cervical precancerous lesions are mostly treated by invasive methods and ablative methods like burning/thermal ablation and cryotherapy-based destruction of abnormal tissue. The excision treatment is also prevalent, which involves large loop excision of the transformation zone (LLETZ) or cold knife excision (CKC) in women who are not eligible for ablative treatment [176,182]. The most effective therapies for HPV-associated cancers are mostly chemotherapy and radiotherapy, which increases overall survivability in the patients. Cisplatin (CDDP) and Carboplatin, platinum-based drugs, are used as chemotherapeutic drugs in cervical cancer. Paclitaxel and docetaxel are commonly used in combination. CDDP induces apoptosis by targeting Bax, Puma, MAPK and BclX family proteins, while Paclitaxel acts as a mitotic drug and stabilizes microtubules [183,184]. A major limitation of chemotherapeutic drugs is the development of resistance to the therapy. Chemotherapy resistance is an outcome of low drug uptake, modulation in hallmarks of cancers like DNA damage, apoptosis and EMT, along with epigenetic modifications related to miRNA expression [184]. miRNA contributes to chemoresistance as well as plays a role in overcoming chemoresistance in therapeutic drugs. For instance, miR-499a contributed to the chemoresistance of CDDP in cervical cell lines, while overexpression of miR-155 increased the chemosensitivity to CDDP [185,186]. Comprehensively, miRNAs can play an important role in diagnosis, treatment and therapeutic strategy to overcome limitations in other modes of treatment.

The miRNAs profoundly regulate gene expressions during carcinogenesis. Multiple clinical trials and studies have been conducted to study the role of miRNAs in their application as biomarkers or as therapeutic targets in treatment. miR-148b acts as a tumor suppressor during cervical cancer by inducing cell cycle arrest in the G1/S phase and apoptosis in a caspase 3-dependent manner, which makes it a prospective therapeutic approach for cervical carcinogenesis [187]. A study by Song et al. identified miR-195 as a potential target for the treatment of cervical cancer [188]; miR-195 inhibits cell proliferation, invasion and migration in cervical cancer, and miR-21, miR-135b, miR-223 and miR-301b are overexpressed in cervical cancer and can be used as biomarkers for cancer progression [189]. The therapeutic capability of miRNA can be employed by the use of mimics and inhibitors. miRNA mimics are small molecules identical to the deregulated miRNA that restore the function of tumor suppressor miRNA, thereby inhibiting the expression of oncogenes. This therapy is termed miRNA replacement therapy. On the other hand, miRNA inhibitors work by silencing the overexpression of oncomiRs and restoring the function of tumor suppressor genes. The therapy involving the use of miRNA inhibitors is termed miRNA inhibitor therapy [65]. The miR-6852 (miR-sX4) exhibited anticancer activity in cervical cancer, while miR-187 was shown to promote apoptosis and inhibition of proliferation in cervical cancer cells in an experimental study on nude mice [190]. Several clinical studies have been undertaken to study the therapeutic potential of miRNA in malignancies. miR-34a has shown promising responses in hepatocellular and renal carcinoma, and studies have reported it leads to the suppression of multiple oncogenes in cervical cancer [189]. miR-21 has been reported to be one of the main targets for the resolution of radiation resistance in cervical cancer [191]. CRISPR-Cas9 gene editing has also proved to be one of the imperative techniques for the knockdown of E6 and E7 oncogenes of HPV, thereby inducing inhibition of cervical cancer [192].

Immunotherapy has also been emerging therapeutics for HPV-associated cancers. E6 and E7 are potential targets for immunotherapy. Approaches like Anti-HPV protein mAbs, radioimmunotherapy, affibodies and affitoxins, single-chain antibodies (scFvs), nanobodies and T-cell-based therapies are novel mechanisms studied for the prevention of HPV-associated cancers. Native Anti-HPV protein monoclonal antibodies are reported to inhibit cervical carcinogenesis in mouse models. Monoclonal antibodies specifically targeting HPV E6 and E749-57 peptides lead to a significant reduction in tumor growth [193]. Radioimmunotherapy involves administering radiolabelled monoclonal antibodies, which bind to specific antigens on tumors. When administered, these mAbs bind to E6 and E7 proteins and deliver cytotoxic radiation resulting in the inhibition of tumor growth. mAbs labeled with Rhenium-188 (a beta emitter) attach to E6, mediating cell death in experimental tumor models. Affibodies are small antibody mimetics that have an affinity towards target proteins and are engineered to bind to a large number of target proteins. Several affibodies target EGFR (Epidermal growth factor receptor), IGFR1 (insulin-like growth factor 1) and epidermal growth factor receptor 2 (HER2). Affitoxins are connected with Pseudomonas toxin, an anti-cancer agent and deliver this toxin with high specificity to tumor cells, making them a promising strategy to combat HPV-induced cancers [194]. Single-chain antibodies and nanobodies are small monoclonal antibodies with high affinity [195]. In the Checkmate trial, Neoadjuvant nivolumab was reported to be safe and induced pathologic regressions in HPV-positive (23.5%) and HPV-negative (5.9%) tumors in HNSCC [196].

Despite the advanced prevention strategies and intervention measures, screening facilities are not available for HPV-associated non-cervical cancers. In a systematic review and meta-analysis conducted by Balachandra et al., the utility and identification of blood-based biomarkers were reviewed [197]. HPV-16 E seropositivity and circulating HPV DNA were found to be strongly correlated with HPV-associated malignancies compared to controls. Cervical cancer was found to be significantly associated with circulating HPV DNA, while the rest of the malignancies, like oropharyngeal, anal and penile cancer, were strongly linked with HPV-16 E seropositivity.

## 12. Conclusions

HPV infection has evolved to be the major cause of multiple malignancies. The malignancies share commonalities in pathways for malignant transformation, tumor biology, response to therapy and biomarkers, which can be helpful in designing proper strategies to combat increasing burden. Multiple treatment strategies are available, including the availability of prophylactic vaccines to the general population. However, vaccine coverage is limited to developed countries and high-income group populations due to high cost, vaccine hesitancy, lack of awareness, and still pending incorporation into universal vaccination programs in many countries. The current vaccines available are only effective if taken before exposure to the virus. However, owing to various disparities in income level, awareness and geographical conditions, it is not available to the majority of the population. WHO recommends that the vaccine be administered at ages 9–15 years. Despite this, there are still high rates of morbidity and incidence rates in various countries. Due to the global increase in HPV infection and even fewer people being administered the vaccine dose, it is imperative that countries prepare specific guidelines for the introduction of vaccines in their vaccination programs, making it cost-effective for high-risk groups and low-income populations. Awareness programs regarding sexually transmitted infections, vaccination coverage, exploring new technologies like miRNA-based therapies and developing strategic plans to implement policies and guidelines with respect to testing, affordable diagnosis and treatment may help in curbing the infection rate and progression in malignancies.

## Figures and Tables

**Table 1:** Unique Features of HPV Positive Malignancies [29,32].

	Significant Feature of HPV -Associated Malignancy	Mechanism Involved	Type of Cancer
HPV Positive Cancers	Enhanced Cell cycle progression	E6 and E7 mediated degradation of p53 and pRb	All
	Increased radiosensitivity	Regulation of wt-p53, accumulation of double-stranded DNA breaks	HNSCC, Cervical cancer, Anal cancer
	Impaired DNA repair	Dysregulation of homologous recombination by the overexpression of p16, Dysregulation of non-homologous end joining by pRB	HNSCC
	Upregulated immune checkpoints proteins like PD-L1 and CTLA-4	E7 mediated PD-L1 expression, Hypermethylation of DNA repair genes	HNSCC, Cervical Cancer
	Altered tumor immune microenvironment	Abundance of CD8+ cells, Increased levels of IL-6, IL-8 and CXCL1	HNSCC, Cervical Cancer
	Accumulation of mutations	Higher APOBEC mutations, Disrupted DNA repair mechanisms	HNSCC, Cervical Cancer
	CpG methylation in both repetitive and non-repetitive regions	Higher Line -1 Methylation and higher genomic instability	HNSCC

## Data Availability

Not applicable.
